# Quality of institution and the FEG (forest, energy intensity, and globalization) -environment relationships in sub-Saharan Africa

**DOI:** 10.1007/s11356-017-9300-2

**Published:** 2017-06-07

**Authors:** Franklin Amuakwa-Mensah, Philip Kofi Adom

**Affiliations:** 1 Department of Economics, Swedish University of Agricultural Sciences (SLU), Uppsala, Sweden; 2 Department of Banking and Finance, University of Professional Studies, Accra, Ghana

**Keywords:** Environmental degradation, Forest cover, Globalization, Energy efficiency, Institutional quality, Sub-Saharan Africa, Q01, Q53, Q55, Q58

## Abstract

The current share of sub-Saharan Africa in global carbon dioxide emissions is negligible compared to major contributors like Asia, Americas, and Europe. This trend is, however, likely to change given that both economic growth and rate of urbanization in the region are projected to be robust in the future. The current study contributes to the literature by examining both the direct and the indirect impacts of quality of institution on the environment. Specifically, we investigate whether the institutional setting in the region provides some sort of a complementary role in the environment-FEG relationships. We use the panel two-step system generalized method of moments (GMM) technique to deal with the simultaneity problem. Data consists of 43 sub-Saharan African countries. The result shows that energy inefficiency compromises environmental standards. However, the quality of the institutional setting helps moderate this negative consequences; countries with good institutions show greater prospects than countries with poor institutions. On the other hand, globalization of the region and increased forest size generate positive environmental outcomes in the region. Their impacts are, however, independent of the quality of institution. Afforestation programs, promotion of other clean energy types, and investment in energy efficiency, basic city infrastructure, and regulatory and institutional structures, are desirable policies to pursue to safeguard the environment.

## Introduction

The increase in global atmospheric concentration of carbon dioxide emissions continues to pose serious threats to the environment and economy at large. The goal of moving towards a low-carbon economy is, thus, very imperative for sustainable economic development, human existence, and environmental sustainability. This goal has motivated the various climate policies implemented worldwide. Though the share of carbon dioxide emissions from sub-Saharan Africa, in global levels, remains very small, the projected rapid economic growth in the region alongside rapid urbanization and industrialization could threaten the goal of a low-carbon economy and subsequently the region’s future environmental and economic sustainability. Therefore, designing the most efficient and cost-effective ways to achieve a low-carbon economy would be imperative going into the future. Africa and sub-Saharan Africa for that matter have been touted to suffer most from climate change problems in the world, and this makes the subject of carbon dioxide emissions very important from an African perspective. The main objective of this study is to investigate the drivers of carbon dioxide emissions, as an indicator for environmental degradation, in sub-Saharan Africa. Specifically, the direct and indirect effects of quality of institution are examined.

The following factors—*forest cover*, *energy intensity, and globalization* (i.e., *foreign direct inflows and trade openness*)—are worth considering from the sub-Saharan African perspective if the region aims at achieving a low-carbon region. The forest can store, capture, and release carbon. The biological growth process of the forest captures carbon from the atmosphere. Also, due to its long life and considerable mass, the forest can hold large volumes of carbon in their cells. Thus, the forest acts as carbon silos, with net biological growth increasing the volumes of carbon held in their cells (see Sedjo and Sohngen (2012)). According to Malhi et al. (2002), forest stores approximately 47% of the global carbon. In residential and urban centers, the forest also provides shades for building, which help reduce the energy required for heating and cooling. This means that increasing the forest cover in the region will generate positive environmental outcomes. The forests in sub-Saharan Africa provide a major source of livelihood both for urban and rural households. According to the World Bank estimates, about 20% of the disposable income of landless and poor families comes from the forest (see World Forest, Society and Environment (WFSE) (2009)). In terms of energy, 85% of the wood removed from the forest and woodlands are used as fuels by rural and urban households (WFSE 2009). The contribution of the forest to gross domestic product is about 10% for 19 African economies and more than 10% contribution in trade for 10 economies (WFSE 2009). The forests in sub-Saharan Africa also provide protection for the environment. It is estimated that forests in the Central Africa store about 25.03 billion tons of carbon. This is equivalent to 4 years of current global anthropogenic emissions of carbon dioxide (WFSE 2009). Also, mature humid forests in Africa sequester 630 kg of carbon per hectare per year, which provides a continual buffer against global climate change. Unfortunately, the forest size in sub-Saharan Africa is declining continuously (see Fig. 2 in Appendix) due to increased need of land for settlement, lack of defined property rights, and lack of enforcement from regulators, among others. This raises serious concern about the future environmental protective role of the forest in the region.

Energy consumption and climate change are closely connected (see Li et al. (2016); Khan et al. (2014); Akhmat et al. (2014a, b); Adom and Amuakwa-Mensah (2016)). Kais and Sami (2016) for a panel of 58 countries, Baek (2016) for ASEAN countries, and Shahbaz et al. (2015a, b) for high-middle- and low-income countries confirm that energy consumption increases carbon dioxide emissions. It is a fact that fossil fuel consumption is a major contributor to global atmospheric concentration of carbon dioxide. According to the Intergovernmental Panel on Climate Change (IPCC), fossil fuel is responsible for 95% of the global carbon dioxide emissions (in Adom et al. (2012)). This explains why governments worldwide advocate for energy efficiency improvements. According to the International Energy Agency (IEA), energy efficiency policies could reduce global carbon dioxide emissions by 10–15%. In China, Gao et al. (2016) note that energy efficiency is the leading force that reduces carbon dioxide emission growth. Energy intensity in sub-Saharan Africa is now more than double the world average and triple the OECD average (see IEA (2014)). This is an indication of how inefficient energy is used in sub-Saharan Africa (SSA). Consequently, carbon dioxide emissions from energy sources are growing in the region (see Adom (2015); Adom and Amuakwa-Mensah (2016)). Given that the population in the region is expected to exceed 1.75 billion by 2040 and economic growth is expected to be robust in the future, there is likely to be a sustained increase in energy use in the future. If unchecked, this is likely to have serious ramifications on the region’s environment and energy security.

The empirical link between environmental performance and foreign direct inflows remains inconclusive. On one hand, foreign direct inflows (FDI) generate negative environmental externality (i.e., pollution haven hypothesis). On the other hand, foreign direct inflows generate positive environmental externality. This makes the issue of FDI-environment relationship a complex issue to investigate. FDI remains an important contributor to economic growth in sub-Saharan Africa. Between 1970 and 2000, the share of FDI as a percentage of gross domestic product averaged 0.939%. But this has increased by more than 100-folds between 2000 and 2014 with an average of 2.701% (calculated by the authors using data from World Development Indicators (WDI)). In 2014, FDI in SSA rose by 5% but this varied by sub-region. While FDI declined by 10% in West Africa and 2% in Southern Africa, it increased by 33% in Central Africa and by 11% in East Africa (see United Nations Conference on Trade and Development (UNCTAD) (2015)). Though growth in FDI has been impressive in recent decades in SSA, it still remains unclear whether FDI offers a protective role for the region’s environment or not. The simple reason is that the FDI-environment relationship is much more complex. The differential impacts of the scale, technical and composition aspects of FDI, make the FDI-environment relationship a complex one. The link between trade openness and environmental performance still remains unclear, and the story is not very different for the sub-Saharan African region. Trade in SSA has improved significantly over the last 20 years. Both export-to-GDP and import-to-GDP ratios have increased from 20.5 and 19% in 1995 to 27.5 and 23% in 2013, respectively (International Monetary Fund (IMF) (2015)). While the positive growth impacts of trade in the region are somewhat obvious, no consensus exists regarding the trade-environment relationship in the sub-region. Depending on the direction of impacts of FDI and trade, a case of environmental quality can be made either in favor or against economic globalization (i.e., financial and trade liberalization). However, this is an empirical question to answer.

From the policy perspective, it is important to know the marginal effects or elasticities of forest cover, energy intensity (proxy for regional energy efficiency), FDI, and trade openness. The marginal effects assign the weights that should be put on public policies aimed at improving environmental performance. In other words, the marginal effects or elasticities help provide a ranking order of importance for forest cover, energy intensity, FDI, and trade openness to guide public policy. There have been earlier attempts that provide the marginal elasticities for energy intensity and globalization (see Al-Mulali et al. (2016); Shahbaz et al. (2015a, b, 2016); Baek (2016); Kohler (2013); Lin et al. (2016); Kais and Sami (2016): Al-Mulali et al. (2015); Keho (2015); Ali et al. (2016); Abid (2016); Jebli et al. (2015); Aboagye and Kwakwa (2014); and Osabuohien et al. (2014)). However, these studies practically assume the impact of these variables to be independent of some regional conditions. Especially from the sub-Saharan Africa perspective, the quality of institutions matters greatly. Good institutions could prevent the pollution haven hypothesis, ensure strict enforcement of regulations to protect the forest, and prevent energy use wastage. In this regard, there are few notable exceptions in the literature: the studies by Osabuohiem et al. (2015) and Ibrahim and Law (2016). Ibrahim and Law (2016) examine the complementary role that institutions play in international trade, while Osabuohiem et al. (2015) investigate the complementary role that institutions play in international trade, FDI, and energy consumption. We are not aware of any study that conditions the impacts of forest cover and energy intensity on the institutional settings in the region. Also, we are not aware of any study that investigates econometrically the environment-forest cover relationship in sub-Saharan Africa, given the economic contribution of the forest to the rural and macro economy. Our study fills these gaps in the literature.

Our contributions are as follows. First, we provide the first empirical-econometric-based estimate of the forest-environment relationship in the sub-region. Second, we condition the impacts of forest cover, energy intensity, and FDI on the quality of institution. One advantage of this approach is that it helps us investigate the complementary environmental protective role played by the institutional settings in the region. We are quick to state that we are not the first to have acknowledged the indirect effects of institution in the discussion of environmental quality in the region. Studies by Ibrahim and Law 2016) and Osabuohien et al. (2014) have both investigated the problem in sub-Saharan and Africa, respectively. In these studies, the authors examined the complementary role of quality of institution via trade openness, foreign direct inflows, and energy consumption. Though the current study follows the same logic adopted in those studies, it differs in some number of ways. First, in addition to FDI, we also estimate the conditional impacts of forest cover and energy intensity. Second, we acknowledge the importance of heterogeneity in such analysis. Therefore we proceed to deduce the heterogeneous marginal conditional impacts of forest cover, energy intensity, and FDI for each country in the sample. Third, we also use the PolityIV measure of democracy as our proxy for institutional quality. This has widely been used in the environment-institution relationship in the literature. However, it has been criticized on the grounds that it only focuses on a section of governance. The Unified Democracy Score (UDS) by Pemstein et al. (2010), which is a more cumulative measure, rather leverages the efforts of several scholars simultaneously. Thus, it offers a more balanced measure for democracy. As a robustness check, therefore, we use the UDS also as a measure of democracy. We estimate a dynamic panel model. Due to possible simultaneity problem that results from the correlation between the lag dependent variable and the error term, we adopt the two-step system panel generalized method of moments (GMM) as our estimation technique. Our sample consists of 43 countries.

The results are as follows. Higher energy intensity worsens environmental quality. However, with good institutional settings in place, the negative consequences of high energy intensity diminishes. Countries with good institutional settings (such as South Africa, Mauritius, Cape Verde, and Botswana) show greater prospects than countries with poor institutional settings (for example, Swaziland and Eritrea). Globalization of the region and increased forest cover enhance environmental performance in the region, but their environmental protective role is independent of the institutional settings, according to our results. Further, we find no support for the environmental Kuznet curve (EKC) hypothesis, and rapid urbanization and intense industrialization do compromise environmental quality in the region.

The remaining sections are structured as follows. “Review of empirical literature: African perspective” reviews the relevant literature. “Model, method, and data” presents the model and discusses the data. “Results and discussion” discusses the results of the study. This section is categorized into two: (1) unconditional impact analyses and (2) conditional impact analyses. “Conclusion” concludes the paper with policy recommendations.

## Review of empirical literature: African perspective

The issue of climate change and how to mitigate its consequences has become very imperative due to the increasing global environmental challenges. In this regard, there have been many empirical attempts in the literature that discuss the different possible ways both economic and political that can help mitigate the adverse effects of climate change. Along this line, studies that focus on the driving forces of carbon dioxide emissions proliferate in the literature for the simple reason that carbon dioxide emissions contribute greatly to climate change. The present study is motivated by similar reason and structured accordingly. Specifically, we review the literature on the growth-environment, globalization-environment, forest-environment, energy-environment, institution-environment, and industrialization-urbanization-environment relationships. However, we focus on studies that investigate the Africa problem; this is to ensure that we put the current study in a proper context.

### Economic growth-environment relationship

Economic growth has been found to be detrimental to the environment, but this, according to the environmental Kuznet curve hypothesis, happens at the early stages of development and reverses at later stages of development. This rationalization has motivated several of the empirical studies that examine the driving forces of carbon dioxide emissions. However, the results are far from unanimity. Al-Mulali et al. (2016) test for the EKC hypothesis for seven regions that include sub-Saharan Africa. Their study controlled for the role of renewable energy consumption. They find no evidence of the existence of the EKC hypothesis. Shahbaz et al. (2016) also test for the EKC hypothesis for selected African countries. Their study controlled for the effect of globalization. Their result shows no evidence of the EKC hypothesis in those countries. Jebli et al. (2015) test for the EKC hypothesis in sub-Saharan Africa. Their study controlled for the effects of renewable energy consumption. Their result shows no evidence of the EKC. Osabuohiem et al. (2015) test for the EKC hypothesis in Africa. Their study controlled for the effects of trade, energy, and foreign direct inflows. Result shows no evidence of the EKC hypothesis. Other studies in the region which found no support for the EKC hypothesis include Lin et al. (2016), Aboagye and Kwakwa (2014), Kohler (2013), and Nasr et al. (2015).

On the contrary, other empirical studies on Africa have established support for the EKC hypothesis (Kais and Sami 2016; Keho 2015; Osabuohien et al. 2014; Shahbaz et al. 2015b). Whereas, Kais and Sami (2016) focused on 58 countries that include sub-Saharan and North African countries, Keho (2015) considered Economic Community of West African States (ECOWAS) countries, and Shahbaz et al. (2015b) also considered selected African countries. Several reasons might explain the mixed position in the literature. These include the empirical structure, study period, and sample size. The conflict in the empirical result contributes to policy confusion in the region. In order to achieve certainty in regional climate policy, several empirical studies are required.

### Economic globalization-environment relationship

The roles of trade liberalization and foreign direct inflows in promoting environmental quality have also been examined. The major motivation has been that economic globalization (i.e., trade liberalization and FDI) is a channel for technological diffusion, which either works to promote or degrade the quality of the environment. Similarly, in this regard, the conclusion is not unanimous in the literature.

Al-Mulali et al. (2016), in a study that covered seven regions including sub-Saharan Africa, find that trade openness worsens environmental quality. Keho (2015) focused on ECOWAS countries and finds that international trade increases environmental degradation. Sharma (2011) also confirms the environmental damaging role of international trade in the region. In contrast, Aboagye and Kwakwa (2014) find that trade liberalization improves environmental quality in sub-Saharan Africa. Shahbaz et al. (2016) also confirms, for selected 19 African countries, that globalization decreases carbon dioxide emissions. Country-specific studies have also confirmed the environmental protective role of trade openness. Ali et al. (2016) find that trade openness reduces carbon dioxide emissions in Nigeria. Adams et al. (2016) find that trade openness decreases environmental degradation in Ghana. Kohler (2013) studies the case of South Africa and concludes that trade openness reduces carbon dioxide emissions. The study by Osabuohien et al. (2014) finds no relationship between trade openness and environmental degradation in Africa. Osabuohiem et al. (2015), however, find that trade openness exerts mixed impacts on the environment for a study that included 27 African countries. They argue that institutional quality helps curtail the adverse effects of international trade. Ibrahim and Law (v) also confirm that the impact of trade openness on environmental degradation depends on the institutional setting in a study that focuses on sub-Saharan Africa. According to the result, trade is harmful to countries with low institutional quality but beneficial to countries with high institutional quality. Since the different dimensions of trade offer differential impacts, the ultimate impact of trade on the environment will depend on the trade position/structure of the economy. Jebli et al. (2015) focus on sub-Saharan Africa and find that while exports increase carbon dioxide emissions, import helps reduce it. Thus, trade is likely to be beneficial for import-oriented economies than export-oriented economies.

The FDI-environment relationship remains debatable in the literature. Though viewed as a major channel for knowledge acquisition, managerial transfer, and technological diffusion, which is expected to promote environmental quality, it has been argued that, for countries where there is no strict environmental regulation, FDI could worsen the environment (i.e., pollution haven hypothesis). Shahbaz et al. (2015a), for example, examined the environmental protective role of FDI for a panel of high-middle- and low-income countries and conclude that FDI increases environmental degradation; supporting the pollution haven hypothesis. Similarly, for a study that focuses on 27 African countries, Osabuohiem et al. (2015) confirm that FDI increases carbon dioxide emissions. However, they argue that with good institutional setting, the environmental damaging effect of FDI is likely to be reversed. On the contrary, Aboagye and Kwakwa (2014) study the case of sub-Saharan Africa and confirm that FDI generates positive environmental outcomes.

### Energy-environment relationship

The relationship between energy consumption and carbon dioxide emissions has been extensively investigated in the literature. From the African perspective, mention can be made from Ali et al. (2016), Shahbaz et al. (2015a, b, 2016), Osabuohiem et al. (2015), Kohler (2013), and Sharma (2011). Ali et al. (2016) estimate the impact of energy consumption on carbon dioxide emissions in Nigeria. Result indicates that energy consumption increases environmental degradation in Nigeria. Shahbaz et al. (2015a) focus on high-middle- and low-income countries and conclude that higher energy consumption compromises environmental quality. Osabuohiem et al. (2015) examine the impact of a type of energy (i.e., electricity) on carbon dioxide emissions in Africa. They conclude that higher electricity consumption increases carbon dioxide emissions. Kohler (2013) focuses on South Africa and also concludes that higher energy consumption worsens the environment. Sharma (2011), in a study that covered 68 countries, also confirms the environmental damaging role of higher energy consumption.

The above results imply that improvement in energy efficiency will generate positive environmental outcomes. To this end, few studies have examined the impact of energy efficiency (i.e., energy intensity) on environmental performance in the region. The notable examples are of Shahbaz et al. (2016) and Shahbaz et al. (2015b). Shahbaz et al. (2016) focus on 19 African countries and conclude that energy intensity increases carbon dioxide emissions. This is confirmed in a similar study by Shahbaz et al. (2015b) that also focuses on African countries.

### Forest cover-environment relationship

The role of the forest is another important driving factor of environmental outcomes in the region. The forest is a carbon sequester (see Englin and Callaway (1995); Zhao et al. (2010); Sedjo and Sohngen (2012)). Zhao et al. (2010) find that, in China, the urban forests play a critical role in offsetting urban carbon dioxide emissions. In Africa, the forest is also a major carbon sequester (see earlier remarks in “Introduction”). However, the increase in deforestation in the region has increased negative environmental outcomes in the region, which include poor rainfall patterns, flooding, and lower agricultural production. Despite the significance of the forest to climate issues in the region, currently, there are no studies that empirically estimate the forest elasticity of environmental performance in the sub-region.

### Institution-environment relationship

The quality of institution and its implications on the quality of the environment have also been examined, albeit evidence in the region is limited. It is believed that good institutions will ensure enforcement and promote good environmental outcomes in the region. Ibrahim and Law (2016) examine the impact of institutional quality on the environment in sub-Saharan Africa. They find that quality of institution provides both direct and indirect benefits to the environment. On its own, institutional quality leads to environmental improvement. Also, it helps moderate the environmental damaging effect of trade openness in the region. Osabuohiem et al. (2015) conduct a similar analysis in Africa and find that the quality of institution helps reverse the environmental damaging effect of FDI, trade openness, and electricity consumption. Osabuohien et al. (2014) also find that institutional quality negatively impacts carbon dioxide emissions. However, the impact is statistically not significant.

### Industrialization-urbanization-environment relationship

African governments continue to pursue the goal of long-term sustainable economic development. In this light, the pursuit of higher industrialization is very central, and there have been policy attempts to promote industrialization in the region. While industrialization promotes sustainable economic development, the environmental outcomes are likely to be negative (see Adom (2015)). This has motivated studies to examine the environmental implications of higher industrialization in Africa. Adom et al. (2012) examine the causal relations between industry structure and carbon dioxide emissions in Ghana, Senegal, and Morocco. They find that industrialization Granger causes carbon dioxide emissions in these countries. Aboagye and Kwakwa (2014) also examine the environmental implications of industrialization for sub-Saharan Africa. They confirm that higher industrialization works at the expense of the environment. Lin et al. (2015) also examine the case of Nigeria but conclude that industrialization does not increase carbon dioxide emissions in the country.

Similarly, the environmental consequences of rapid urbanization have been examined in the literature, but the conclusion is not unanimous. Aboagye and Kwakwa (2014) find that rapid urbanization worsens the environment in sub-Saharan Africa. Adams et al. (2016) also conclude that rapid urbanization worsens the environment in Ghana, but this is moderated by an improvement in the quality of institutions. Al-Mulali et al. (2016) confirm the positive impact of urbanization on carbon dioxide emissions, but they find the impact not to be statistically significant. Similarly, Ali et al. (2016) confirm that urbanization has no significant impact on the environment. In contrast, Kais and Sami (2016), for a study that included sub-Saharan Africa, conclude that urbanization reduces carbon dioxide emissions in the region. Similarly, Sharma (2011) finds that urbanization reduces carbon dioxide emissions in the region.

### Literature gaps

We identify the following potential gaps in the above literature, which the current study seeks to address. First, there is no study that econometrically and empirically examines the forest-environment relationship in the sub-region. Given that forest is a major economic event in the region, this represents a major lacuna in the literature. In this study, we estimate the marginal effect of forest cover on environmental outcome in sub-Saharan Africa. Since the institutional setting in the region may matter, we also condition the effect of forest cover on the quality of institution in the region.

Second, regarding the energy intensity-environment relationship, only the studies by Shahbaz et al. (2016) and Shahbaz et al. (2015b) provide empirical evidence for sub-Saharan Africa. We follow the examples of these studies but with two main modifications. First, in addition to the aggregate measure of energy intensity adopted in these studies, we use disaggregate measures like petroleum energy intensity and electricity energy intensity. This we believe provides a more balance view of the energy efficiency-environment relation. An important observation, which is ignored in the present literature, is the role that institutional setting plays in moderating the environmental damaging effect of higher energy intensity. In an environment where the institutional setting is poor, energy efficiency policies are likely to be compromised. The consequence is that instituting energy efficiency will not produce the desirable environmental outcome. However, the story is likely to be different if the quality of institution is better. The current study investigates this issue by conditioning the impact of the energy efficiency indicator on the institutional setting in the region.

Third, the literature on the role of institutional quality in the globalization-environment relationship is scanty for the sub-region. The notable examples are those of Ibrahim and Law (2016) and Osabuohiem et al. (2015). We contribute to this extant literature by conditioning the impact of FDI on the quality of institution. Fourth, it is a fact that different countries will exhibit different dynamics regarding the environment-FDI-forest-energy intensity relationship. Focusing on the pooled effect without taking into these heterogeneities will compromise the beauty of the results. In contrast to Ibrahim and Law (2015) and Osabuohiem et al. (2015), we proceed to display in a graphical form the heterogeneous conditional impacts of FDI, energy intensity, and forest cover.

Fifth, studies that have analyzed the complementary role of institutional quality via different means have done so using different indicators of democracy, most notably the Freedom House and PolityIV measure of democracy. These measures are criticized on the grounds that they are not cumulative in nature and focus on some dimensions of institution. As a robustness check, we use the Unified Democracy Score (UDS) which is a cumulative score that leverages the efforts of several scholars simultaneously. This we believe provides a solid ground for our empirical findings.

Finally, in terms of the model framework, our empirical model is more exhaustive to the extent that the existing empirical frameworks in the literature on Africa can be considered as a microcosm of our model. Therefore, in terms of robustness to omitted variable bias, our model provides a much balanced result.

## Model, method, and data

### Theoretical model

Theoretically, the environmental impact can be decomposed into three component parts: population, consumption/affluence, and technology. Mathematically, this relationship can be written out as Eq. 1, where *I* denotes the environmental impacts, *C* is consumption per head in aggregate terms, *P* is population, and *T* represents technology of consumption (and production).

1 $$ I= P\times C\times T $$

The environmental impacts can be measured in a variety of ways. These include physical units such as the amount of a resource utilized, or of a pollutant released, or an area of land degraded. Consumption could be proxied by gross domestic product per capita, which is the sum of consumption (excludes subsistence agriculture and hence does not account for the associated environmental impacts such as deforestation), government, and investment expenditures. Population could also be proxied by different indicators of population dynamics such as the total population, the population density, and the rate of urbanization. Technology in some cases may reflect the inputs of production, the disposal of waste, and the process of transforming production and consumption. In this regard, economic globalization (i.e., trade openness and foreign direct inflows) and energy efficiency, which ensures energy is not wasted, are important proxies. Economic globalization facilitates transfer of capital, knowledge, and managerial skills across economies; this helps change the input composition and the knowledge process of transforming production and consumption.

It could also indicate, in other cases, the social arrangements, which include the effectiveness of the legal systems and governance, and definition of property rights. In this respect, the quality of institution does matter, since it ensures enforcement of enactment that deters bad practices and defines property rights, which creates ownership and prevent public abuse use of resources. Using the appropriate proxies, the above equation can be reformulated as Eq. 2, where PD is population density; GDP is gross domestic product per capita; FC is forest cover to capture the environmental impacts of subsistence agriculture, which is captured in GDP; FD is foreign direct investment; TO is trade openness; EI is energy intensity (a surrogate for energy efficiency); Inst is the quality of institution; and UR is rate of urbanization.

2 $$ I=\mathrm{PD}\times \mathrm{GDP}\times \mathrm{FC}\times \mathrm{FD}\times \mathrm{TO}\times \mathrm{EI}\times \mathrm{Inst}\times \mathrm{UR} $$

### Empirical model

Our empirical model is based on Eq. 2; however, we rationalize it based on the environmental Kuznet curve (EKC). The EKC hypothesizes that environmental degradation increases in a non-linear form with income/economic growth. This has informed many empirical papers that seek to understand the driving forces of environmental degradation (see Shahbaz et al. (2016); Baek (2016); Kohler (2013); Al-Mulali et al. (2015); Keho (2015); Kais and Sami (2016); Osabuohien et al. (2014); Jebli et al. (2015); Osabuohiem et al. (2015); Al-Mulali et al. (2016)). However, since this relation is conditioned on other factors, empirical studies on the subject have controlled for other important variables. This has been the main differential point for many of these studies. Our empirical model also hinges on the rationalization of the EKC hypothesis. However, our set of controlled variables differs from the other empirical attempts in the literature. We specify environmental degradation (measured as carbon dioxide emissions) as a multiplicative function of real GDP per capita (*Y*), square of real GDP per capita (*Y* ^2^), forest cover (FC), energy intensity (EI), foreign direct inflows (FD), trade openness (TO), industry value added (IV), urbanization (UR), population density (PD), and quality of democracy (Inst). The inclusion of industry value added is to account for the effects of structural changes. We have also included the lag of environmental impacts to account for persistence. Since we are interested in the marginal effects, we posit a Cobb-Douglas relationship. This is shown in Eq. 3. Log transformation of Eq. 3 produces Eq. 4. Note that a non-zero assumption is imposed on the regressors.

3 $$ {\mathrm{C}\mathrm{O}}_2={{\mathrm{C}\mathrm{O}}_{2\mathrm{t}-1}}^{\alpha}{Y}^{\beta}{Y}^2{}^{\beta_2}\mathrm{F}{\mathrm{C}}^{\chi}{\mathrm{EI}}^{\delta}{e}^{{\mathrm{FD}}^{\varphi}}{e}^{{\mathrm{TO}}^{\gamma}}{\mathrm{IV}}^{\theta}{e}^{{\mathrm{UR}}^{\varpi}}{\mathrm{PD}}^{\uppi}{e}^{{\mathrm{Inst}}^{\psi}} $$

4 $$ \ln \mathrm{CO}{2}_{2\mathrm{t}}=\alpha \ln {\mathrm{CO}}_{2\mathrm{t}-1}+{\beta}_1 \ln {Y}_{\mathrm{t}}+{\beta}_2 \ln {Y_{\mathrm{t}}}^2+\chi \ln {\mathrm{FC}}_{\mathrm{t}}+\delta \ln {\mathrm{EI}}_{\mathrm{t}}+\varphi {\mathrm{FD}}_{\mathrm{t}}+\gamma {\mathrm{TO}}_{\mathrm{t}}+ \ln {\mathrm{IV}}_{\mathrm{t}}+\varpi {\mathrm{UR}}_{\mathrm{t}}+\pi \ln {\mathrm{PD}}_{\mathrm{t}}+\psi {\mathrm{Inst}}_{\mathrm{t}}+{\varepsilon}_{\mathrm{t}} $$

Equation 4 shares some similarities with other empirical specifications in the literature. Aboagye and Kwakwa (2014) in their study controlled for urbanization, industrialization, trade openness, and foreign direct inflows. Their empirical model is a microcosm of our empirical specification. Jebli et al. (2015) also controlled for trade openness. However, unlike the current study, they also controlled for the effect of renewable energy. Comparably, the current model controls for a lot of other variables that minimizes the problem of omitted variable bias, which is possible with the study by Jebli et al. (2015). Osabuohien et al. (2014) in quiet a similar way controlled for the effects of institutional quality and trade openness. Thus, their study can be regarded as a microcosm of our model. Keho (2015) controlled for the effects of trade openness and population. Similar to Osabuohien et al. (2014), Keho (2015) is a miniature of the current study. Kais and Sami (2016) and Shahbaz et al. (2014) in their study controlled for energy consumption, urbanization, and trade openness. Our model captures the role of energy efficiency more appropriately than Kais and Sami (2016). Also, their study can be regarded as a microcosm of the current study. Kohler (2013) also controlled for the effects of foreign direct inflows and energy consumption. Shahbaz et al. (2016) had energy intensity and globalization as controls in their model. The empirical models adopted by Kohler (2013) and Shahbaz et al. (2016) are a microcosm of our empirical specification. Finally, Al-Mulali et al. (2016) controlled for renewable energy consumption, trade openness, urbanization, and domestic credit to the private sector. In terms of model generalization, our model is more general than other studies in the region. This makes the current model more robust to the omitted variable bias problem. There are other studies that examine similar problem in Africa. These include Adom et al. (2012), Mensah (2015), Ibrahim and Law (2016), Ali et al. (2016), and Lin et al. (2015). However, these studies do not hinge on the EKC rationalization. Therefore, the current study deviates from the above based on this ground.

Since the environmental protective role of the forest, foreign direct inflows, and energy intensity may be dependent on the institutional setting in the region, we condition the impacts of these variables on the quality of democracy in the sub-region. In this regard, we interact the institution variable with each of these variables and include them as additional covariates in the model. Note that we do this systematically. In this setup, our model is comparable to the empirical models estimated by Ibrahim and Law (2016) for sub-Saharan Africa and Osabuohiem et al. (2015) for 27 African countries. Ibrahim and Law (2016) control for the effects of trade, institution, urbanization, money supply, and credit to private sector. They then interact the trade variable with the institution variable. We do otherwise in the current study. We condition the impact of forest cover, energy intensity, and foreign direct inflows on the institutional settings. Osabuohiem et al. (2015) control for trade openness, foreign direct inflows, and quality of institution. However, in contrast to their study that controlled for electricity consumption, we control for aggregate energy intensity, petroleum energy intensity, and electricity intensity. In this regard, our model captures the effect of energy efficiency more appropriately than their study. Further, while we examine the complementary role that institutions offer to the forest, foreign direct inflows, and energy intensity, Osabuohiem et al. (2015) make the impact of trade openness, foreign direct inflows, and electricity consumption conditional on the institutional settings. Equation 5 is the conditional model we estimate, where *X* ^*i*^ is the *i*th variable to be conditioned (*i* = FD, FC, and EI).

5 $$ \ln \mathrm{CO}{2}_{2\mathrm{t}}=\alpha \ln {\mathrm{CO}}_{2\mathrm{t}-1}+{\beta}_1 \ln {Y}_{\mathrm{t}}+{\beta}_2 \ln {Y_{\mathrm{t}}}^2+\chi \ln {\mathrm{FC}}_{\mathrm{t}}+\delta \ln {\mathrm{EI}}_{\mathrm{t}}+\varphi {\mathrm{FD}}_{\mathrm{t}}+\gamma {TO}_{\mathrm{t}}+ \ln {\mathrm{IV}}_{\mathrm{t}}+\varpi {\mathrm{UR}}_{\mathrm{t}}+\pi \ln {\mathrm{PD}}_{\mathrm{t}}+\psi {\mathrm{Inst}}_{\mathrm{t}}+{\theta}_i{X^i}_{\mathrm{t}}\times {\mathrm{Inst}}_{\mathrm{t}}+{\varepsilon}_{\mathrm{t}} $$

Equations 4 and 5 are reformulated as dynamic panel models in Eqs. 4b and 5b, where *μ* and *λ* indicate country-specific effects and time-specific effects, respectively. The country-specific effects account for the influence of unobserved variables such as culture, history, and infrastructure, while the time-specific effects account for the time-varying omitted variables and the stochastic shocks which are common to all countries.

4b $$ \ln \mathrm{CO}{2}_{2 i\mathrm{t}}=\alpha \ln {\mathrm{CO}}_{2 i\mathrm{t}-1}+{\beta}_1 \ln {Y}_{i\mathrm{t}}+{\beta}_2 \ln {Y_{\mathrm{t} i}}^2+\chi \ln {\mathrm{FC}}_{i\mathrm{t}}+\delta \ln {\mathrm{EI}}_{i\mathrm{t}}+\varphi {\mathrm{FD}}_{i\mathrm{t}}+\gamma {\mathrm{TO}}_{i\mathrm{t}}+ \ln {\mathrm{IV}}_{i\mathrm{t}}+\varpi {\mathrm{UR}}_{i\mathrm{t}}+\pi \ln {\mathrm{PD}}_{i\mathrm{t}}+\psi {\mathrm{Inst}}_{i\mathrm{t}}+{\mu}_{i\mathrm{t}}+{\lambda}_{i\mathrm{t}}+{\varepsilon}_{i t} $$

5b $$ \ln \mathrm{CO}{2}_{2 i\mathrm{t}}=\alpha \ln {\mathrm{CO}}_{2 i\mathrm{t}-1}+{\beta}_1 \ln {Y}_{i\mathrm{t}}+{\beta}_2 \ln {Y_{i\mathrm{t}}}^2+\chi \ln {\mathrm{FC}}_{i\mathrm{t}}+\delta \ln {\mathrm{EI}}_{i\mathrm{t}}+\varphi {\mathrm{FD}}_{i\mathrm{t}}+\gamma {\mathrm{TO}}_{i\mathrm{t}}+ \ln {\mathrm{IV}}_{i\mathrm{t}}+\varpi {\mathrm{UR}}_{i\mathrm{t}}+\pi \ln {\mathrm{PD}}_{i\mathrm{t}}+\psi {\mathrm{Inst}}_{i\mathrm{t}}+{\theta}_i{X^i}_{i\mathrm{t}}\times {\mathrm{Inst}}_{i\mathrm{t}}+{\mu}_{i\mathrm{t}}+{\lambda}_{i\mathrm{t}}+{\varepsilon}_{i\mathrm{t}} $$

We measure democracy using the PolityIV measure. Obviously since this measure is not cumulative in nature, we run the risk of only focusing on some aspects of governance at the expense of the other aspects. This problem is very peculiar to all previous studies that have used some form of an indicator mostly from Freedom House and PolityIV, which is not cumulative in nature. In other to circumvent around this problem, we introduce another measure of democracy, the UDS, which leverages the efforts of several scholars simultaneously; this is used as a robustness check.

### Method: econometric issues and estimation

There are two fundamental econometric problems associated with Eqs. 4b and 5b, and that makes the traditional approaches such as the pooled OLS, fixed and random effects methods inappropriate techniques to apply in this case. The first has to do with the correlation between the lagged dependent variable and the error term and between the unobserved country-specific effects and the lagged dependent variable. The second is the possibility of endogeneity of some of the explanatory variables. Arellano and Bond (1991) propose the one-step general method of moments differencing method to solve this problem. Their method involves using a set of internal instruments. However, the approach by Arellano and Bond (1991) suffers from lack of power of the internal instruments. In the case where the lags of the dependent and explanatory variables exhibit persistence, the lag of the levels of these variables become weak instruments for the GMM equation in differences (see Blundell and Bond (1998)); this introduces bias and imprecision.

The two-step GMM helps reduce this bias and imprecision. In contrast to the one-step GMM, the weights specified to the level estimators, in the two-step GMM, grow in the prevalence of weak instruments due to the high persistence in the series (Jaunky 2013). The first stage of the two-step GMM assumes an independent and homoscedastic error terms. In the second stage, the method uses the first-step residuals to construct consistent variances and covariance matrices. This makes the two-step GMM more robust to serial correlation and heteroscedasticity. Based on these reasons, we employ the two-step system GMM as the estimation technique. Though this approach is more efficient than the one-step GMM, the technique converges slowly to its asymptotic distribution. Consequently, its standard errors suffer from downward bias, for finite samples. Windmeijer (2005) suggests a finite-sample correction to the system GMM. The approach recommends the use of multiple lags as instruments, and this results in an over-identification problem. Thus, we do acknowledge that the system GMM does has a weakness. To address this concern, we minimize the number of lags and employ the Sargan’s test to check for the over-identification restrictions. This is important to establish the validity of the instruments. Further, we test for autocorrelation in the errors. There is no autocorrelation in the errors if the errors for the first differenced equations confirm autocorrelation at order 1and no autocorrelation at order 2.

### Data

The study uses panel data covering 43 sub-Saharan Africa countries. Data for the model with aggregate energy intensity covers 1990 to 2011, while data for the model with disaggregate energy intensity covers 1990 to 2012. Table 1 shows the variables, descriptive statistics, and data source. The mean FDI is 3.50; this deviates from the actual value by 8.28. The high variability is an indication of high uncertainty in foreign direct inflows in the region. A host of factors including the unstable nature of the economic structure in the region, which causes investor panic in the region, might explain this outcome. For example, elections have been ragged in the region several times causing political instability in the region. Similarly, the high uncertainty in economic policies and growth in the region also cause investor panic in the region and that could explain the high variability in FDI. Also, the shift in economic focus by external economies which causes redistribution of FDI from one region to another might explain this phenomenon. The distribution of FDI is normal and positively skewed. The mean level of trade integration is high (i.e., 70) with a standard deviation of 34.6. The data is positively skewed and normally distributed. Average rate of urbanization is 33.86 with a standard deviation of 14.89. Data is normally distributed and positively skewed. The average quality of institution is a little above 0.5 which indicates moderate institutional improvement in the region. The standard deviation is 0.28. The distribution is non-normal, and it is positively skewed. Average aggregate energy intensity^1^ is 7.805 with a standard deviation of 0.806. The high mean level indicates high incidence of energy inefficiency in the region. The distribution is normal and negatively skewed. Average per capita income is 6.39, which is US$596.45, with a deviation of 0.99, which is US$2.7. Data is normally distributed and positively skewed. Per capita CO_2_ averages^2^ at −1.495 with a deviation of 1.299. The distribution is normal, and data is positively skewed. Average industrialization^3^ is 3.127 with a standard deviation of 0.514. The distribution is normal, and data is negatively skewed. Population density^4^ averages at 3.568 with a standard deviation of 1.311. Data is negatively skewed, and the distribution is not normal. Average petroleum^5^ and electricity intensity^6^ are −12.98 and 15.44 with a standard deviation of 0.623 and 0.830, respectively. The distribution is normal, and data is positively skewed. The average forest size^7^ is 10.59 with a standard deviation of 2.2. The distribution is normally distributed, but the data is negatively skewed. Thus, the left tail of the distribution is longer than the right tail of the distribution.

**Table 1:** Descriptive statistics

Variables	Definition	Source	(1) N	(2) Mean	(3) SD	(4) Min	(5) Max	(6) Skewness	(7) Kurtosis
FDI	Foreign direct investment, net inflows (% of GDP)	WDI^a^	932	3.503	8.275	−82.89	91.01	3.46	51.40
Trade	Trade volume (% of GDP)	WDI	923	69.94	34.57	10.75	209.9	1.21	4.42
Urbanization	Urban population (% of total)	WDI	946	33.86	14.89	5.416	86.05	0.63	3.61
Institution^b^	Proxied by polity2. The polity score is computed by subtracting the p_autocracy score from the p_democracy score	Polity IV Project^c^	946	0.536	0.280	0	1	0.04	1.64
lnei	Natural log of energy intensity (ratio of energy consumption to GDP)	EIA^d^	942	7.805	0.806	5.188	10.28	−0.004	3.37
lnGDPPC	Natural log of real GDP per capita	WDI	943	6.391	0.993	3.913	8.940	0.88	3.17
lnCO_2_	Natural log of CO_2_ emissions (metric tons per capita)	WDI	881	-1.495	1.299	−5.586	2.309	0.57	3.34
lnforest	Natural log of forest area (sq. km)	WDI	943	10.59	2.204	3.258	14.29	−0.96	3.50
Insti × forest	Institution multiplied by forest		943	5.587	3.001	0	11.23	0.17	1.84
GDPPCsq	Squared of lnGDPPC		943	41.83	13.61	15.31	79.92	1.17	3.60
lnIVA	Natural log of industry, value added (% of GDP)	WDI	916	3.127	0.514	0.632	4.349	−0.17	3.56
lnpopden	Natural log of population density (people per sq. km)	WDI	945	3.568	1.311	0.542	6.425	−0.21	2.65
lnei × Insti	Energy intensity multiplied by institution		942	4.215	2.287	0	9.111	0.15	1.77
lneipet	Petroleum intensity (ratio of total petroleum consumption to GDP)	EIA	1007	−12.98	0.623	−15.47	−10.63	0.06	5.43
lneielec	Electricity intensity (ratio of total electricity net consumption to GDP)	EIA	1007	−15.44	0.830	−17.93	−12.90	0.29	3.55

*EIA* Energy Information Administration, *WDI* World Development Indicators

^a^ http://data.worldbank.org/data-catalog/world-development-indicators

^b^Polity2 scale ranges from +10 (strongly democratic) to −10 (strongly autocratic); we, however, transformed the variable to range from 0(strongly autocratic) to 1(strongly democratic) for easy interpretation

^c^ http://www.systemicpeace.org/polity/polity4.htm

^d^ http://www.eia.gov/petroleum/data.cfm

## Results and discussion

This section presents and discusses the main results of the study. It begins with a preliminary analysis of the data and then proceeds to analyze the unconditional and conditional drivers of carbon dioxide emissions. Under the unconditional impact analysis, the underpinning assumption is that the impact of the variables that we examine is independent of any regional specific conditions. Under the conditional impact analysis, we relax this assumption and condition the impacts of forest cover, energy intensity, and foreign direct inflows on the institutional quality in the region.

### Unit root test

Table 2 shows the unit root test for the selected variables. The panel Augmented Dickey-Fuller and Phillip-Perron tests are used in this study. These methods are known to account for individual unit root process. Therefore, in dealing with heterogeneity, it is preferred over unit root tests that assume a common unit root process. All two tests by Augmented Dickey-Fuller (ADF) and Phillip-Perron (PP) confirm that carbon dioxide emission is stationary in levels. Similar result is obtained for forest cover, petroleum energy intensity, electricity energy intensity, foreign direct inflows, industry value as percent of GDP, population density, trade openness, quality of institutions, and the interactions between institutional quality, forest cover, energy intensity, and foreign direct inflows. For energy intensity, the ADF-based test confirms stationarity at levels, but the PP-based test provides conflicting results. While the inverse logit-based test confirms non-stationarity of the series in levels, the modified inverse chi-square confirms stationarity of series in levels. Regarding real GDP per capita and the square of real GDP per capita, both tests offer contradictory results. The ADF-based test concludes that both variables are stationary in levels, but the PP-based test concludes that these variables only become stationary after first difference. Since the PP test has more power than the ADF test, we difference the series to achieve stationarity. Since the focus is on the economic growth-environment relationship, we can interpret the associated growth coefficient as the economic growth elasticity. Thus, technically, our series are all stationary in levels.

**Table 2:** Unit root test

	ADF		Philip-Perron	
	Inverse logit	Modified inv. chi-square	Inverse logit	Modified inv. chi-square
lnCO_2_	−11.18***	15.1***	−2.72***	6.058***
lnGDPPC	−3.844***	6.486***	4.594	−0.362
ΔlnGDPPC	−26.77***	42.06***	−24.34***	38.53***
lnGDPPCsq	−3.518***	6.371***	4.792	−0.072
ΔlnGDPPCsq	−26.58***	41.73***	−23.98***	37.92***
lnforest	−6.466***	19.074***	−22.90***	19.424***
lnei	−10.34***	13.76***	−0.82	1.92**
lneipet	−11.18***	14.66***	−1.867**	3.268***
lneielec	−10.05***	13.403***	−0.632	3.171***
FDI	-15.21***	22.655***	−7.30***	12.165***
lnIVA	−12.33***	16.50***	−3.468***	4.537***
Urbanization	5.121	21.73***	−22.14***	27.61***
lnpopden	−10.18***	34.48***	−27.32***	44.20***
Trade	−13.43***	18.43***	−5.468***	7.99***
Institution	−19.65***	29.95***	−16.84***	26.96***
Insti × forest	−22.498***	34.464***	−19.09***	31.96***
Insti × EI	−21.54***	33.04***	−17.35***	28.697***
Insti × FDI	−13.44***	20.44***	−5.95***	9.67***

*p < 0.1; **p < 0.05; ***p < 0.01

### Unconditional impacts of forest cover, FDI, and energy intensity

Table 3 shows the baseline results. Models 1 and 2 include aggregate energy intensity, while models 3 and 4 include petroleum energy intensity and electricity energy intensity, respectively. The rationale is to check whether our model is sensitive to the type of energy intensity measure used. Secondly, we examine how our model result is sensitive to the inclusion of the institutional quality variable. Model 1 shows that carbon dioxide emissions are persistent. The lag coefficient of carbon dioxide emissions is positive and statistically significant. Similar result is obtained even when we include the institutional quality variable. This result is also robust to the different energy intensity measures. Models 3 and 4 confirm that carbon dioxide emissions exhibit inertia. Our result does not confirm the EKC hypothesis. Both models 1 and 3 show that carbon dioxide emissions increase monotonically with economic growth. However, the significance of this relationship disappears when we adjust the model to include the institutional quality variable. This is evident in models 2 and 4. Previous studies on Africa such as those of Lin et al. (2016), Jebli et al. (2015), Nasr et al. (2015), Shahbaz et al. (2016), and Al-Mulali et al. (2016) did not find support for the existence of the EKC in Africa. However, Osabuohien et al. (2014) and Shahbaz et al. (2015a) find support for the EKC in Africa.

**Table 3:** Baseline result

	Aggregated energy intensity		Disaggregated energy intensity	
Variables	(1) No institution	(2) Institution	(3) No institution	(4) Institution
L.lnCO_2_	0.609***	0.555***	0.636***	0.571***
	(0.0471)	(0.0336)	(0.0507)	(0.0387)
dlnGDPPC	−2.456***	−1.004	−1.764	−1.663
	(0.519)	(1.634)	(1.211)	(1.560)
dGDPPCsq	0.239***	0.118	0.181*	0.173
	(0.0466)	(0.141)	(0.103)	(0.134)
lnforest	−0.130***	−0.0996**	−0.118***	−0.0870**
	(0.0291)	(0.0409)	(0.0392)	(0.0415)
lnei	0.227***	0.197***	–	–
	(0.0250)	(0.0232)		
lneipet	–	–	0.0894***	0.0289
			(0.0262)	(0.0254)
lneielec	–	–	0.0847***	0.0838***
			(0.0214)	(0.0245)
FDI	−0.00207***	−0.00247***	−0.00105*	−0.00143**
	(0.000514)	(0.000715)	(0.000538)	(0.000684)
lnIVA	0.144***	0.180***	0.119***	0.180***
	(0.0234)	(0.0234)	(0.0210)	(0.0212)
Urbanization	0.0232***	0.0251***	0.0252***	0.0268***
	(0.00333)	(0.00287)	(0.00337)	(0.00265)
lnpopden	−0.0146	−0.00822	−0.0338	−0.0198
	(0.0399)	(0.0370)	(0.0482)	(0.0418)
Trade	−0.000744***	−0.000524**	−0.000438**	−0.000583**
	(0.000256)	(0.000253)	(0.000208)	(0.000261)
Institution	–	−0.0599*	–	−0.0619**
		(0.0328)		(0.0310)
Constant	−2.040***	−2.406***	2.191***	0.690
	(0.420)	(0.549)	(0.520)	(0.596)
Observations	812	792	812	792
No. of countries	43	42	43	42
Wald chi^2^	49,717.83***	48,816.28***	44,844.7***	11,625.4***
Sargan’s test (S)	32.94 (37)	31.06 (37)	33 (37)	31.8 (37)
First-order autocor.	−2.05**	−1.97**	−2.06**	−2.02**
Second-order autocor.	−1.29	−1.21	−1.27	−1.23
No. instruments	48	49	49	50

Standard errors are in the parentheses. In the Sargan’s test, we presented the values and the degrees of freedom in the parentheses. We presented the *z* values for the autocorrelation test

*p < 0.1; **p < 0.05; ***p < 0.01

The impact of forest cover on carbon dioxide emissions is negative and statistically significant as shown in model 1. This result remains robust even when we include the institutional quality variable. Similar result is obtained when we use the petroleum energy intensity and electricity intensity to replace the aggregate energy intensity measure both for the model with and without the institutional quality variable. For the models that use the aggregate measure of energy intensity, carbon dioxide emissions are expected to decrease by between 1.3 and 1% for every 10% increase in forest cover in sub-Saharan Africa. For the models that use petroleum and electricity energy intensity, carbon dioxide emissions are expected to decrease by between 1.18 and 0.87% for every 10% increase in the size of the forest cover. With the gradual erosion of the forest in the sub-region, our results suggest that carbon dioxide emissions will continue to increase if nothing is done to reverse the current trend in the forest sector in the region. The inelastic nature of carbon dioxide emissions to forest cover, however, implies that the region cannot solely rely on the improvement of the forest cover as a remedy to the increasing carbon dioxide emissions. For a greater impact, other complementary programs have to be rolled over.

The impact of aggregate energy intensity on carbon dioxide emissions is significantly positive. It is estimated that for every 10% increase in energy intensity, carbon dioxide emissions will increase by 2.27% (see model 1). When we control for the quality of institutions, the impact of energy intensity is still statistically significant and positive. According to the estimate, a 10% increase in energy intensity will cause carbon dioxide emissions to increase approximately by 2%. The impacts of petroleum energy intensity and electricity energy intensity on carbon dioxide emissions, as shown in models 3 and 4, are also significantly positive. According to the estimate, a 10% increase in either petroleum energy intensity or electricity energy intensity will cause an increase in carbon dioxide emissions by 0.89 and 0.85%, respectively. However, only electricity energy intensity is able to retain its robustness after we control for the quality of institutions in the sub-region. The positive energy intensity elasticity is confirmed in the studies by Shahbaz et al. (2016) for selected African countries, Lin et al. (2015) for Nigeria, and Shahbaz et al. (2015a) for selected African countries. However, Song et al. (2015) find that in China, energy intensity constraints carbon emissions. With the continuous growth in the region’s energy intensity, carbon dioxide emissions will continue to increase and threaten the sustainability of the environment and economy. Since energy intensity is a rough surrogate for energy efficiency, our results imply that improving upon energy efficiency in the sub-region will significantly help reduce carbon dioxide emissions. However, the inelastic nature of the effect means that this should be complemented with other programs to be able to realize a greater impact on environmental performance.

Foreign direct inflows significantly reduce carbon dioxide emissions in the sub-region. Model 1 shows that a 10 percentage point increase in foreign direct inflows will cause carbon dioxide emissions to decline by 2.07%. The result remains robust when we use the disaggregate measures of energy intensity. As shown in model 3, the impact of foreign direct inflows is significantly negative, though the size of the impact is smaller. A 10 percentage point increase in foreign direct inflows is expected to cause carbon dioxide emissions to decline by 1.07%. For both cases, when we control for the quality of institutions in the region (i.e., models 2 and 4), the impact of FDI is still significantly negative. The estimates show that a 10 percentage point increase in foreign direct inflows will cause carbon dioxide emissions to decrease by 2.47 and 1.43%. In contrast to the current study’s finding, Shahbaz et al. (2015b) for high-middle- and low-income countries and Osabuohiem et al. (2015) for 19 African countries find FDIs to increase carbon dioxide emissions. However, Aboagye and Kwakwa (2014) confirm the negative impact of FDI on the environment in sub-Saharan Africa.

Trade openness also has a significant negative impact on carbon dioxide emissions. The result remains robust in all cases. The negative impact ranges from 0.00044 to 0.00074. This implies that for every 10 percentage point increase in trade openness, carbon dioxide emissions will fall by between 0.044 and 0.074%. In congruence with the current result, Kohler (2013) for South Africa, Aboagye and Kwakwa (2014) for sub-Saharan Africa, and Ali et al. (2016) for Nigeria find that trade openness reduces carbon dioxide emissions. Jebli et al. (2015) use a decomposed measure of openness and find that export/GDP increases carbon dioxide emissions but imports/GDP reduces carbon dioxide emissions in sub-Saharan Africa. Osabuohien et al. (2014) find a negative trade openness effect but statistically insignificant in Africa. In contrast to the current study, Al-Mulali et al. (2016) for seven regions and Keho (2015) for ECOWAS find that trade openness worsens environmental degradation. While Ibrahim and Law (2016) for sub-Saharan Africa argued that the trade-environment relationship is influenced by the institutional settings, Osabuohiem et al. (2015) find a mixed result for Africa. According to the current result, both financial liberalization (i.e., FDI) and trade liberalization are eco-friendly. The negative impacts of both FDI and trade liberalization, thus, imply that globalization of the sub-region is eco-friendly. This is consistent with the findings of Shahbaz et al. (2016) for selected African countries.

An increase in the rate of industrialization compromises the quality of the environment. The result shows that increasing the rate of industrialization by 10% will cause carbon dioxide emissions to increase by 1.44%, according to model 1. Similar result is obtained when we use the disaggregate measure of energy intensity instead of the aggregate measure. The elasticity is estimated as 0.119, according to model 3. The result remains robust when we control for the quality of institutions in the region. The elasticity is estimated as 0.180. The positive impact of industrialization implies that an enhanced industrialization is not eco-friendly in the sub-region. The simple reason is that the industrial sector is the most energy-intensive sector in the region. Importantly, however, the effect is inelastic, which means that carbon dioxide emissions will increase by less than proportionately. Thus, it is expected that if the growth returns of industrialization is huge, the sub-region, on the net, should be better off. Contrary to our results, Lin et al. (2015) find that industrialization in Nigeria does not lead to higher carbon dioxide emissions.

Increased urbanization is not eco-friendly. The result shows that urbanization significantly increases carbon dioxide emissions in the sub-region. The result remains robust when we use the disaggregate measure of energy intensity and control for the quality of institutions. The estimate ranges from 0.0232 to 0.0268. Thus, an increase of 10 percentage point in urbanization will cause carbon dioxide emissions to increase by 23.2 and 26.8%. Urbanization in sub-Saharan Africa has failed to create an inclusive growth. Rather, it has contributed to slum proliferation in the sub-region. In Africa, sub-Saharan Africa has the lowest urban population (about 32.8%) but the highest slum dwellers (about 65%). This is because the region has poor urban settlement arrangements and lacks basic infrastructures. About 20% of the population in SSA has access to electricity and about 60% live in places with inadequate water supplies and sanitation. The proliferation of slums in the region has contributed to increased pollution in the sub-region. Secondly, the rapid urbanization has increased the pressure on natural resources and the environment. Expansion of cities has led to the destruction of forest and other natural environment. Thirdly, the rapid urbanization has contributed to increased energy consumption, and since energy consumption is closely connected to environmental pollution, environmental pollution has increased in the sub-region. Urban population growth is predicted at 3.5%, and it is expected to persist into 2050. Based on the result, it is expected that the quality of the environment would be compromised if proper urban planning and investment in basic infrastructures are not pursued. The result that urbanization compromises environmental quality is confirmed by Al-Mulali et al. (2015) and Li et al. (2016), which focused on the European region and Chinese economy, respectively, and Adams et al. (2016), which focused on Ghana. For Nigeria, Ali et al. (2016) find that the impact of urbanization is insignificant. However, in a study by Kais and Sami (2016) that focused on 58 countries that included sub-Saharan Africa, it is evident that urbanization is eco-friendly.

The quality of institutions has a significant positive implications for the environment. According to the result, improved institution significantly causes carbon dioxide emissions to decrease. The result remains robust irrespective of the measure of energy intensity used. According to the estimate, 10 percentage point increase in institutional quality will cause carbon dioxide emissions to fall by 60–62%. Developed countries are known to have strict regulations regarding the environment, which serve as a greater disincentive to environmentally unfriendly firms. These firms are forced to relocate to developing countries that have loose regulations (pollution haven hypothesis). The result suggests that with good institutions in place, these environmentally unfriendly firms will not find the region as the safe haven. Ibrahim and Law (2016) also find that institutional quality improves the quality of the environment in sub-Saharan Africa. Adams et al. (2016) also confirmed that good institutions promote clean environment in Ghana. Though Osabuohien et al. (2014) find the impact of institution to be negative, it is statistically not significant. The impact of population density on carbon dioxide emissions is negative but statistically not significant. The diagnostic statistics for the various estimations pass the Sargan’s test of instrument validation and the second-order autocorrelation test.

### Conditional impact of forest cover, FDI, and energy intensity

In this section, we condition the impact of forest cover, FDI, and energy intensity on the quality of institution in the region. We approach this step systematically. First, we include the interaction of forest cover and quality of institution into the model. In subsequent regressions, we include the interactions of energy intensity and quality of institution and the interactions of FDI and quality of institutions in separate regressions. Model 1 in Table 4 shows the result when we include the interaction of forest cover and quality of institutions. The impact of economic growth on carbon dioxide emissions increases monotonically, though statistically, it is not significant. Higher energy intensity, intense industrialization, and rapid urbanization leads to significant environmental degradation. The result shows that the impact of these variables is significantly positive. An increase in energy intensity by 10% is expected to increase carbon dioxide emissions by approximately 2.1%. The industrialization elasticity is estimated as 0.148, which implies that, with a 10% increase in industrialization, carbon dioxide emissions will increase by 1.48%. Similarly, an increase of 10 percentage points in urbanization will cause carbon dioxide emissions to decrease by 29%. These results confirm the baseline result, though with a small deviation. There is no significant relationship between population density and carbon dioxide emissions and between trade openness and carbon dioxide emissions. The interaction of forest cover and quality of institutions is statistically not significant. This means that the impact of forest cover on carbon dioxide emissions is independent of the quality of the institutions. However, the direct effect of forest cover reduces to −0.101. Thus, an increase of 10% in forest cover will cause carbon dioxide emissions to decline by 1.01%.

**Table 4:** Conditional effect

Variables	(1) Forest	(2) EI	(3) FDI
L.lnCO_2_	0.517***	0.542***	0.557***
	(0.0427)	(0.0393)	(0.0344)
dlnGDPPC	−2.255	−2.529	−0.707
	(1.619)	(2.070)	(1.653)
dGDPPCsq	0.226	0.246	0.0920
	(0.140)	(0.178)	(0.143)
lnforest	−0.101**	−0.0708*	−0.0952**
	(0.0477)	(0.0420)	(0.0401)
lnei	0.205***	0.365***	0.198***
	(0.0318)	(0.0854)	(0.0265)
FDI	−0.00251***	−0.00258***	−0.00222
	(0.000732)	(0.000680)	(0.00168)
lnIVA	0.148***	0.176***	0.182***
	(0.0327)	(0.0217)	(0.0257)
Urbanization	0.0289***	0.0251***	0.0244***
	(0.00375)	(0.00314)	(0.00329)
lnpopden	0.00839	0.0298	−0.000980
	(0.0381)	(0.0449)	(0.0378)
Trade	−0.000295	−0.000393	−0.000544**
	(0.000352)	(0.000248)	(0.000274)
Institution	−0.279	2.469*	−0.0568
	(0.667)	(1.321)	(0.0363)
Insti × forest	0.0197	–	–
	(0.0602)		
Insti × EI	–	−0.325*	–
		(0.170)	
Insti × FDI	–	–	−0.000249
			(0.00441)
Total effect of Insti	–	−0.072	–
		(0.045)	
Total effect of EI	–	0.191***	–
		(0.0281)	
Constant	−2.603***	−4.159***	−2.461***
	(0.638)	(1.102)	(0.548)
Observations	792	792	792
No. of countries	42	42	42
Wald chi2	53,324.38***	33,285.61***	41,489.16***
Sargan’s test (S)	28.73 (37)	30.82 (37)	30.69 (37)
First-order autocor.	−1.96**	−2.04**	−1.97**
Second-order autocor.	−1.244	−1.271	−1.205

Standard errors are in the parentheses. In the Sargan’s test, we presented the values and the degrees of freedom in the parentheses. We presented the *z* values for the autocorrelation test

*p < 0.1; **p < 0.05; ***p < 0.01

Model 2 shows the result when we include the interaction of energy intensity and quality of institutions. The impact of economic growth on carbon dioxide emissions increases monotonically. However, this is statistically not significant. Increasing the forest cover and the inflow of FDI is eco-friendly. The results show that both impact negatively on carbon dioxide emissions. For forest cover, an increase of 10% will cause a decline in carbon dioxide emissions by 0.71%. On the other hand, an increase of 10 percentage points in FDI will decrease carbon dioxide emissions by 2.58%. On the contrary, higher industrialization and urbanization are not eco-friendly. The results indicate that both increase carbon dioxide emissions in the sub-region. These results are also consistent with the baseline results, but the size of the coefficients differs slightly. The impact of trade and population density is not significant. The interaction between energy intensity and quality of institutions is significant and negative. This means that the impact of energy intensity on carbon dioxide emissions is dependent on the quality of institutions. Without good institutions in place, energy efficiency policies can easily be compromised, but good institutions ensure that energy efficiency policies are enforced. The total effect of energy intensity is significantly positive, but the results show that with improvement in the quality of institutions, this positive impact is likely to be reduced and helps improve the quality of the environment.

**Fig. 1:**
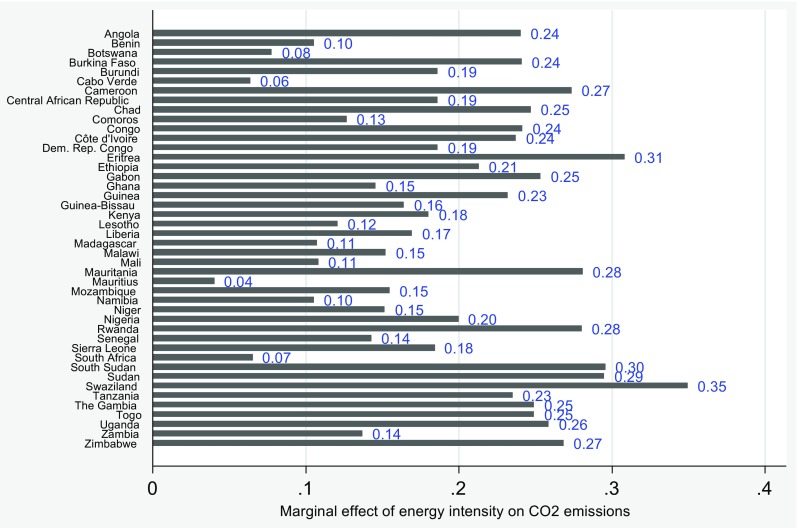
Marginal effect of energy intensity on carbon dioxide emissions

**Fig. 2:**
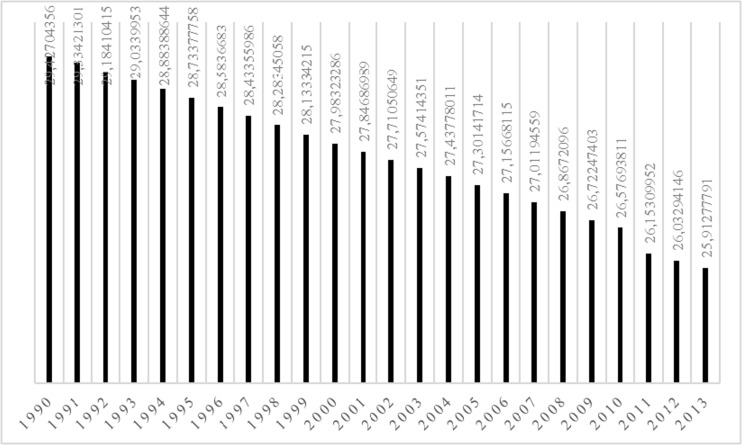
Forest cover as percentage of total land area in sub-Saharan Africa. Data source: World Development Indicators

Since the total effect is based on the group average, it is difficult to delineate the actual country effects of energy intensity on carbon dioxide emissions. Figure 1 shows the marginal effects of energy intensity on carbon dioxide emissions for each country in the sample. There is a significant heterogeneity in the marginal elasticities of energy intensity. South Africa, Mauritius, Cape Verde, and Botswana have the lowest marginal elasticity values. It ranges from 0.04 to 0.08. Swaziland (0.35) and Eritrea (0.31) have the highest marginal elasticity values. The marginal elasticities for Zambia, Sierra Leone, Niger, Namibia, Mozambique, Mali, Malawi, Madagascar, Liberia, Lesotho, Kenya, Nigeria, Guinea-Bissau, Ghana, Democratic Republic Congo, Comoros, Central Africa Republic, and Burundi range from 0.1 to 0.2, while the remaining countries’ marginal elasticities range from 0.2 to 0.3. The size of these elasticity values are greatly influenced by the quality of institutions in these economies. South Africa, Mauritius, Cape Verde, and Botswana rank among the most stable democracies in the region. Mauritius has an overall government score (this includes all other indicators of institutions) of 81.7 out of the 100; Cape Verde has a score of 76.6; Botswana has a score of 76.2; and South Africa has a score of 73.3 (according to 2014 governance score). Therefore, in these economies, the quality of institutions helps moderate the sensitivity of carbon dioxide emissions to energy intensity changes. According to the current elasticities, carbon dioxide emissions are less sensitive to energy intensity changes. Swaziland is a non-democratic state, and Eritrea is ranked at the bottom with a score of 29.8 out of 100. The poor democratic nature of these economies helps increase the sensitivity of carbon dioxide emissions to energy intensity changes. Thus, in these economies, the probability that energy efficiency policies can easily be compromised is very high. The rest of the countries that fall within the different elasticity bounds have quasi-developed institutions, which help moderate the sensitivity of carbon dioxide emissions to energy intensity changes. For example, Namibia, Lesotho, Ghana, Comoros, and Mali with scores of 70.3, 62.3, 68.2, 49.3, and 49.5 fall within a lower elasticity bound compared to Togo, Ethiopia, Cameroon, Angola, and Zimbabwe with scores of 46.4, 48.5, 47.6, 40.9, and 38. The implication of the result is that energy inefficiency will cause environmental degradation but with good institutions in place that ensures strict enforcement of regulations, the negative environmental consequences of energy inefficiency will diminish. The total effect of institutions conditioned on energy intensity is not significant though negative.

Finally, model 3 shows the conditional impact of foreign direct inflows. Carbon dioxide emissions increase monotonically with economic growth, but this is statistically not significant. An increase in forest cover and trade openness significantly improves the quality of the environment. On the contrary, energy inefficiency, intense industrialization, and rapid urbanization significantly contributes to environmental degradation. These results are consistent with earlier results obtained in this study. The interaction between FDI and institution is negative but statistically insignificant. This means that the impact of FDI and institution is independent of each other. Thus, both variables move in parallel paths. The various models were subjected to some diagnostic tests. All the models pass the autocorrelation and Sargan’s test of instrument validation.

### Robustness check

We estimated the same models using the UDS measure of democracy. As argued earlier, this measure leverages the efforts of several scholars simultaneously and solves the problem of focusing on some dimensions of institution. According to model 1, in Table 5, carbon dioxide emissions exhibit inertia; this confirms the earlier result. There is no evidence of the EKC hypothesis, which also confirms previous findings. Forest cover, FDI, trade openness, and institution improve environmental quality; however, the effect of forest is found to be insignificant in this case. On the contrary, urbanization, industrialization, and energy intensity worsen environmental quality. These results confirm our earlier results. The results also remain robust when we use the disaggregate measure of energy intensity. The conditional models also remain robust except that the indirect effects, albeit retained their a priori expectation in the case of energy intensity and FDI, become insignificant. In all, we conclude that the results remain robust when we the UDS measure of democracy.

**Table 5:** Carbon dioxide estimations using UDS as proxy for institution

Variables	(1) Institution	(2) Disaggregation	(3) Forest	(4) EI	(5) FDI
L.lnCO_2_	0.564***	0.607***	0.507***	0.547***	0.564***
	(0.0380)	(0.0426)	(0.0449)	(0.0457)	(0.0373)
dlnGDPPC	−0.721	−1.424	−2.090	−2.007	−0.472
	(1.481)	(1.385)	(1.491)	(1.703)	(1.487)
dGDPPCsq	0.0956	0.157	0.212	0.204	0.0746
	(0.127)	(0.117)	(0.129)	(0.147)	(0.128)
lnforest	−0.0465	−0.149***	−0.138**	−0.0831*	−0.0478
	(0.0577)	(0.0372)	(0.0555)	(0.0453)	(0.0565)
lnei	0.203***		0.164***	0.298***	0.207***
	(0.0327)		(0.0306)	(0.0971)	(0.0347)
lneipet		0.0779***			
		(0.0275)			
lneielec		0.0794***			
		(0.0209)			
FDI	-0.00237***	−0.00155**	−0.00203***	−0.00289***	−0.000244
	(0.000709)	(0.000607)	(0.000744)	(0.000780)	(0.00102)
lnIVA	0.166***	0.122***	0.182***	0.174***	0.170***
	(0.0278)	(0.0251)	(0.0289)	(0.0280)	(0.0281)
Urbanization	0.0238***	0.0276***	0.0284***	0.0254***	0.0242***
	(0.00285)	(0.00296)	(0.00350)	(0.00376)	(0.00292)
lnpopden	0.00919	−0.0485	−0.0476	0.0116	0.0166
	(0.0368)	(0.0467)	(0.0457)	(0.0488)	(0.0353)
Trade	−0.000566**	−0.000302	−0.000360	−0.000399	−0.000523*
	(0.000281)	(0.000206)	(0.000295)	(0.000261)	(0.000284)
Institution	−0.0875*	−0.147***	−1.191	1.865	−0.0751
	(0.0503)	(0.0446)	(0.861)	(1.815)	(0.0519)
Institution × forest			0.103		
			(0.0796)		
Institution × EI				−0.254	
				(0.237)	
Institution × FDI					−0.00676
					(0.00461)
Constant	−2.965***	2.275***	−1.797**	−3.424***	−3.034***
	(0.687)	(0.576)	(0.730)	(1.200)	(0.664)
Observations	792	812	792	792	792
No. of countries	42	43	42	42	42
Wald chi2	26,414.49***	27,526.88***	117,794.68***	57,541.25***	30,986.54***
Sargan’s test (S)	30.15 (37)	33.75 (37)	28.54 (37)	31.41 (37)	29.38 (37)
First-order autocor.	−1.93*	−2.06**	−1.92*	−2.00**	−1.92*
Second-order autocor.	−1.26	−1.30	−1.26	−1.27	−1.24

*p < 0.1; **p < 0.05; ***p < 0.01

## Conclusion

This study examined the drivers of carbon dioxide emissions as a measure of an environmental quality outcome in sub-Saharan Africa. As a contribution, the study examined the environmental protective roles of the forest, FDI, and energy intensity and the complementary role that the quality of institutions plays in ensuring environmental quality. The panel two-step system GMM was used as the estimation technique with data covering 43 sub-Saharan African countries.

First, we find no support for the EKC hypothesis. Environmental degradation seems to increase monotonically with economic growth, but this relationship disappears once we control for the quality of institutions. Second, increasing the size of the forest cover is environmentally friendly. However, the marginal contribution implies that solely relying on policies that improve the forest cover can only move the environmental quality frontier a bit but cannot overturn the whole situation completely. Further, the results show that the environmental protective role of the forest, in this case, is independent of the quality of institution.

Third, energy intensity has a significant positive impact on carbon dioxide emissions in SSA. This is also true when we use disaggregate measures. Thus, improving upon energy efficiency would generate positive environmental outcomes. However, the small elasticity value suggests that other complementary programs may be required in order to generate greater positive environmental impacts. According to the result, the quality of institutions greatly matters in this case. The result shows greater heterogeneity with countries with good institutions (i.e., South Africa, Botswana, Cape Verde, and Mauritius) showing greater prospects than countries with a quasi-developed institution (i.e., Zambia, Sierra Leone, Niger, Namibia, Mozambique, Mali, Malawi, Madagascar, Liberia, Lesotho, Kenya, Nigeria, Guinea-Bissau, Ghana, Democratic Republic Congo, Comoros, Central Africa Republic, and Burundi) and poorly developed institutions (i.e., Swaziland and Eritrea).

Fourth, foreign direct inflows significantly enhance the quality of the environment, but we found that the environmental protective role of FDI in the region is independent of the quality of institutions. Similarly, trade openness significantly improves environmental performance. The positive environmental externality associated with financial liberalization and trade openness suggests that economic globalization of the sub-region will generate positive environmental outcomes.

Last, the quality of institutions significantly promotes good environmental outcomes. In this case, it causes significant reduction in carbon dioxide emissions. However, intense industrialization and rapid urbanization significantly worsen environmental outcomes. They both lead to higher carbon dioxide emissions. Further, robustness check using the UDS measure of democracy confirms these results.

The above results have the following policy implications. Afforestation programs should be encouraged, and deforestation acts should be discouraged. This will save the forest and help provide the safety net for the environment. Changing the energy mix and promoting energy efficiency should be pursued aggressively to improve the quality of the environment. In this regard, investment in renewable energies and energy efficiency is crucial to the call. Economic globalization should be encouraged and pursued aggressively. However, this should be on the background of a strongly developed institution and legal systems in order to prevent problems like the pollution haven hypothesis. Changing the structure of the economy from the more energy intensive sector to the less energy intensive sector could help moderate the negative environmental impact of output expansion. However, this may be at the expense of long-term sustainable development. Promoting cleaner energy types and investing in energy efficiency in the industrial sector could help achieve both economic and environmental sustainability. Investing in energy efficiency should not be seen as an isolated program. Rather, it must complement the development of strong institutions in various sectors of the economy. Investment in basic city infrastructure and proper urban planning could also help decrease urban pollution significantly.

## Footnotes

1 Energy intensity is in log

2 Per capita CO_2_ is in log

3 In log

4 In Log

5 In log

6 In log

7 In log
